# Key concepts for informed health choices: Where’s the evidence?

**DOI:** 10.12688/f1000research.123051.2

**Published:** 2023-11-27

**Authors:** Andrew D. Oxman, Iain Chalmers, Astrid Dahlgren

**Affiliations:** 1 Centre for Epidemic Interventions Research, Norwegian Institute of Public Health, Oslo, Norway; 2 Centre for Evidence-Based Medicine, Department of Primary Care, University of Oxford, Oxford, UK; 3 Department of Nursing and Health Promotion, Faculty of Health Sciences, Oslo Metropolitan University, Oslo, Norway

**Keywords:** concepts, critical thinking, critical appraisal, causal inference, critical health literacy, treatment claims, informed decision making, epistemology; systematic review

## Abstract

**Background:**

The Informed Health Choices (IHC) Key Concepts is a framework that provides a basis for developing educational resources and evaluating people’s ability to think critically about health actions. We developed the original Key Concepts framework by reviewing texts and checklists for the public, journalists, and health professionals and collecting structured feedback from an international advisory group. We revised the original 2015 framework yearly from 2016 to 2018 based on feedback and experience using the framework. The objectives of this paper are to describe the development of the framework since 2018 and summarise their basis.

**Methods:**

For the 2019 version, we responded to feedback on the 2018 version. For the current 2022 version, in addition to responding to feedback on the 2019 version, we reviewed the evidence base for each of the concepts. Whenever possible, we referenced systematic reviews that provide a basis for a concept. We screened all Cochrane methodology reviews and searched Epistemonikos, PubMed, and Google Scholar for methodology reviews and meta-epidemiological studies.

**Results:**

The original framework included 32 concepts in six groups. The 2019 version and the current 2022 version include 49 concepts in the same three main groups that we have used since 2016. There are now 10 subgroups or higher-level concepts. For each concept, there is an explanation including one or more examples, the basis for the concept, and implications. Over 600 references are cited that support the concepts, and over half of the references are systematic reviews.

**Conclusions:**

There is a large body of evidence that supports the IHC key concepts and we have received few suggestions for changes since 2019.

## Introduction

At the end of the last century, at least 28 frameworks were published describing the need for competencies and curriculum changes for primary and secondary schools in the 21 ^st^ century. ^1^ Since then, many countries have moved or are moving from knowledge-based to competence- or skill-based primary and secondary school curricula. ^2^ Critical thinking is one of the most often included competences. ^1^^–^^3^

Critical thinking is not a new idea. It dates at least to Socrates over 2,400 years ago, and the need to teach critical thinking in school has been argued for over 100 years. ^4^^,^^5^ There are many different definitions of critical thinking, ^5^ and debate about how it should be taught. ^6^ However, generally, the focus is on dispositions and competences that help people to decide what to believe or do.

A major new challenge to deciding what to believe or do is increased access to information online and in social media, and the need to evaluate that information, much of which is misinformation. ^4^ A huge amount of health information can be found online, in addition to information that is disseminated through other channels of communication. This problem has been exacerbated by the COVID-19 pandemic, which has been accompanied by an ’infodemic’ – an overload of information including false or misleading information. ^7^

In the context of health, the skills needed to decide what to believe or do are sometimes referred to as critical health literacy. ^8^^,^^9^ Although both critical thinking and health are widely included in primary and secondary school curricula, critical thinking about health or critical health literacy may not be, ^10^^–^^13^ and many people find it difficult to make decisions about what to believe or do regarding ’treatments’ or ’health actions’ (things that they can do to care for their health or the health of others). ^14^

Being able to understand and apply some basic principles or concepts is essential for using reliable information appropriately and avoiding being misled by unreliable information. This includes concepts about claims, comparisons (research evidence from treatment comparisons), and choices ( Table 1). As noted by Dewey, ^15^ *“*it would be impossible to overestimate the educational importance of arriving at conceptions: that is, of meanings that are general because applicable in a great variety of different instances in spite of their difference; that are constant, uniform, or self-identical in what they refer to, and that are standardized, known points of reference by which to get our bearings when we are plunged into the strange and unknown.” ^16^

**Table 1.** Three main groups of concepts and 10 high-level concepts in the 2022 version of the Informed Health Choices (IHC) Key Concepts. ^18^

1. Claims	2. Comparisons	3. Choices
*Claims about effects that are not supported by evidence from fair comparisons are not necessarily wrong, but there is an insufficient basis for believing them.*	*Studies should make fair comparisons, designed to minimize the risk of systematic errors (biases) and random errors (the play of chance).*	*What to do depends on judgements about a problem, the relevance of the available evidence, and the balance of expected benefits, harms, and costs.*
1.1 Assumptions that treatments are safe or effective can be misleading. 1.2 Seemingly logical assumptions about *research* can be misleading. 1.3 Seemingly logical assumptions about *treatments* can be misleading. 1.4 Trust based on the source of a claim alone can be misleading.	2.1 Comparisons of treatments should be fair. 2.2 Reviews of the effects of treatments should be fair. 2.3 Descriptions of effects should clearly reflect *the size of the effects.* 2.4 Descriptions of effects should reflect *the risk of being misled by the play of chance.*	3.1 Evidence should be relevant. 3.2 Expected advantages should outweigh expected disadvantages.

The Informed Health Choices (IHC) Network has developed a framework that includes 49 key concepts as a starting point for deciding what to teach. ^17^^,^^18^ The framework provides a basis for developing learning and teaching resources and evaluating learners’ ability to think critically about health actions. Most of the concepts in the framework are relevant to other types of actions or interventions, including agricultural, educational, and environmental interventions. ^19^

The concepts serve as the basis for developing learning resources to help people understand and apply the concepts when claims about the effects of health actions are made, and when they make health choices. ^20^ They are also the basis for an item bank of multiple-choice questions (the Claim Evaluation Tools item bank) that can be used for assessing people’s ability to apply the IHC Key Concepts. ^2^

The concepts are principles for recognising when a claim about the effects of health actions has an untrustworthy basis, recognising when evidence from comparisons of health actions is trustworthy and when it is not, and making well-informed health choices. They can help anyone, not just researchers, to think critically about whether to believe a claim and what to do. The concepts are intended for people using research, not for doing research.

The concepts and the basis for the concepts tend to focus on ways in which claims and comparisons (evidence) can be misleading, and choices can be misinformed. This is not because we underestimate the tremendous gains that have been made in health care based on appropriate use of research. Our aim is to promote healthy scepticism, not excessive scepticism, and to help people decide what to believe and what to do. How sure we can be about the effects of health actions varies, as does how sure we can be about the balance between the advantages and disadvantages of health actions. When teaching, learning, or using these concepts, it is important to bear this in mind.

Although most people prefer to make medical decisions together with a health professional, some people prefer to delegate decisions to a health professional. ^21^ Unfortunately, health professionals’ perceptions of their patients’ desire to be involved in decisions are often inaccurate. ^22^ They may be more likely to underestimate the extent to which patients prefer to be involved in decisions. Regardless of who decides, decisions should be consistent with a patient’s values. Decision aids can help patients to clarify their values and may help them to make choices that are more consistent with their values compared to choices made without decision aids. ^23^ There is some evidence that patients choose more conservative approaches when they become better informed. ^24^

Even people who prefer to delegate medical decisions to a health professional are confronted with an over-abundance of information about things they can do for their health, much of which is unreliable. ^7^^,^^25^^–^^30^ Although much of this information can simply be ignored, to avoid being misled by claims with an untrustworthy basis, people need to be able to recognise claims about the effects of health actions, question the basis for those claims, and recognise when a claim is relevant and important, and warrants reflection.

People have the right to participate collectively as well as individually in the planning and implementation of their health care. ^31^ In addition to being a democratic right, public participation in deliberative and decision-making processes has the potential to improve the quality of the judgements and decisions that are made, build trust, improve adherence, and help to ensure transparency and accountability. ^32^ However, the extent to which potential benefits of public participation are achieved (and potential harms are avoided) depends, among other things, on the ability of citizens to think critically about collective decisions as well as personal choices.

People often disagree about the effects of health actions, including experts. Who makes a claim, how likable they are, or how much experience and expertise they have do not provide a reliable basis for assessing how reliable their claim is. This does not mean that conflicting opinions should be given equal weight – or that the existence of conflicting opinions means that no conclusion can be reached. It also does not mean that all sources of information are equally reliable. How much weight to give an opinion should be based on the strength of the evidence supporting it. How much trust to place in a source of information about health actions should be based on whether it provides information about the effects of health actions based on systematic reviews. ^25^

The objective of this paper is to describe the development of the IHC Key Concepts from 2019 to 2022 and to summarise the development of the framework over the past decade, its basis, and how it has been used.

### Development of the Informed Health Choices Key Concepts 2013-2018

We have previously summarized development of the IHC Key Concepts up to the 2018 version. ^17^ Development of the IHC Key Concepts started in 2013 as the first step in a five-year research project funded by the Research Council of Norway (GLOBVAC project 220603/H10). ^33^ The objectives of this project were to develop and evaluate resources for primary schools and mass media to improve people’s ability to assess claims about the effects of treatments. We began by extracting all the concepts addressed in *Testing Treatments*, ^34^ a book that was written to promote more critical public assessment of claims about the effects of treatments. We then searched the literature for other relevant material, including books and checklists for the public, journalists, and health professionals. ^35^ Our aim has been to include all concepts that are important for people to consider, while minimising redundancy.

Initially, we collected structured feedback from an international advisory group using four questions: ^35^

1. Are concepts included that should not be?
2. Are there important concepts that are missing?
3. Are the concepts organised in a logical way?
4. Do you have any other comments regarding the concepts?

We published the first version of the list in 2015. ^35^ That list included 32 concepts in six groups. Subsequently, we collected feedback at a series of workshops in 2017 and 2018, ^17^ and from colleagues working in other fields besides health in 2018. ^19^^,^^36^ We have used four criteria in deciding on changes to the list of concepts. ^17^ New key concepts must:

• be within the scope of the IHC Key Concepts – standards for judgment, or principles for evaluating the trustworthiness of treatment claims and treatment comparisons (research) used to support claims, and to inform treatment choices,
• address ways in which treatment claims and comparisons are frequently misleading or ways in which poorly informed decisions are taken,
• be useful for people without a research background to use research, not just for researchers or for doing research, and
• overlap as little as possible with other key concepts.

In addition, revisions have been informed by a review of related frameworks in 2018, ^37^ and using the concepts to:

• systematically review the effects of educational interventions to improve people’s understanding of the key concepts, ^38^^,^^39^
• create a database of educational interventions to improve people’s understanding of the key concepts, ^40^
• develop and evaluate educational resources. ^10^^–^^12^^,^^41^^–^^53^
• develop an item bank and outcome measures with multiple-choice questions that assess an individual’s understanding of and ability to apply the key concepts, ^54^^–^^59^
• develop a plain language glossary of evaluation terms, ^60^
• ensure coverage of an international core curriculum for teaching evidence-based health care to health professional students, ^20^^,^^61^
• survey the public’s ability to assess treatment claims, ^14^ and
• systematically review the quality of information in news reports about the effects of health interventions. ^62^

We published revised versions yearly from 2016 to 2018. The 2016 version included 34 concepts in three groups, and the 2017 version included 36 concepts. The 2018 version included 44 concepts reorganised within each of the three main groups, and we added three subgroups (higher-level concepts) to each of the three main groups. This helped to clarify the logic behind how the concepts were organised and helped to make the long lists of concepts less overwhelming.

## Methods

The following invitation was included in the 2018 and 2019 versions: Please send any comments or suggestions to: contact@informedhealthchoices.org. In addition, we asked people to provide feedback at workshops where we presented the Key Concepts. Seven individuals provided feedback on the 2018 version and four provided feedback on the 2019 version. The suggestions came from members of the IHC Network and other researchers.

For the 2019 version, we reviewed and responded to feedback on the 2018 version. For the current, 2022 version, in addition to reviewing and responding to feedback on the 2019 version, ^17^^,^^63^ we reviewed the evidence base for each of the concepts. For each concept, we have provided one or more examples to illustrate the explanation, and the basis for the concept. The examples, for the most part, were taken from relevant methodological research. Due to the nature of the research, most of the examples are medical. We selected examples that clearly illustrate the concept and that can easily be understood with little explanation by a wide audience without a medical background.

Whenever possible, we have referenced systematic reviews that provide a basis for a concept. We started with reviews with which we were familiar, including some that we had co-authored. Additional systematic reviews were identified by searching and screening the following sources:

• All Cochrane methodology reviews ^64^ (n = 36, on 29 June 2021)
• Epistemonikos ^65^ using the terms “methodology review” OR “meta-epidemiological” in the title or abstract (n = 161, on 11 October 2021)
• PubMed using the terms “methodology review” OR “meta-epidemiological” (n = 193, on 11 October 2021)
• Google Scholar using the terms “methodology review” OR “meta-epidemiological” in the title, restricted to “Review articles” (n = 370, on 11 October 2021)

We did not restrict searches or exclude reviews based on language or the date of publication.

In addition, we searched Epistemonikos, PubMed, and Google Scholar for systematic reviews that support the explanation for each concept using key terms or phrases from the explanation or related terms. These searches were conducted and screened by one of the authors (ADO). The searches varied for each concept. For example, when our initial searches did not find a recent systematic review, we used citation searches in Google Scholar to search for a recent review or more recent research. We did not record the searches that were conducted for each concept. The purpose of these searches was to summarise the evidence supporting each concept, in addition to the logical explanations.

We included systematic reviews of methodological studies and overviews of reviews, such as Cochrane methodology reviews, methodological studies based on a systematic review of research (“meta-epidemiological” studies), and systematic reviews of treatment effects. We categorised as systematic reviews any review of research that included a methods section that described the search strategy for finding studies and selection criteria.

### Ethical considerations

This research was undertaken as part of two larger projects funded in part by the Research Council of Norway (Project numbers 220603/H10 and 284683). Because the projects will not generate new knowledge about health and disease, approval by the Regional Committee for Medical Research Ethics in Norway was not required, as confirmed by the committee (reference number 30713).

The only people who participated directly in this research were people who provided feedback on earlier versions of the IHC Key Concepts framework. That feedback was given voluntarily with the understanding that it would be used to improve the framework.

## Results

The 2019 version of the framework included 49 concepts. ^63^ We added five new concepts in response to feedback:

• Assumptions that fair comparisons are not relevant can be misleading.
• Your own prior beliefs may be wrong.
• Consider the baseline risk or the severity of the symptoms when estimating the size of expected effects.
• Consider how important each advantage and disadvantage is when weighing the pros and cons.
• Important uncertainties about the effects of treatments should be reduced by further fair comparisons.

We organised the concepts in the same way as in the 2018 version of the framework, under three higher-level concepts in each of the three main groups ( Table 2).

**Table 2.** Overview of revisions to the Informed Health Choices (IHC) Key Concepts.

Version	Groups	Subgroups (higher-level concepts)	Concepts	Competences	Dispositions
**2015** **^35^**	1. Recognising the need for fair comparisons of treatments. 2. Judging whether a comparison of treatments is a fair comparison. 3. Understanding the role of chance 4. Considering all the relevant fair comparisons. 5. Understanding the results of fair comparisons of treatments. 6. Judging whether fair comparisons of treatments are relevant.		32		
**2016** **^66^**	1. Claims 2. Comparisons 3. Choices		34		
**2017** **^67^**			36		
**2018** **^68^**		1. **Beware of treatment claims like these** 1.1 Beware of claims that are too good to be true. 1.2 Beware of claims based on faulty logic. 1.3 Beware of claims based on trust alone. 2. **Check the evidence from treatment comparisons** 2.1 Don’t be misled by unfair comparisons. 2.2 Don’t be misled by unreliable summaries of treatment comparisons. 2.3 Don’t be misled by how treatment effects are described. 3. **Make well-informed treatment choices** 3.1 What is the problem and what are the options? 3.2 Is the evidence relevant? 3.3 Do the advantages outweigh the disadvantages?	44	10	10
**2019** **^63^**			49	20 *	15 *
**2022** **^18^**		( Table 1)	49	20	15

* The competences and dispositions were reformulated and reorganised, as well as expanded.

In response to feedback, ^69^ we also edited the list of concepts in the 2019 version of the framework to make their descriptions more consistent, and we edited some of the explanations. We also clarified our goal ( Table 3), increased the list of competences needed to achieve that goal from 10 to 20, and increased the list of dispositions from 10 to 15.

**Table 3.** Goal of the Informed Health Choices (IHC) Key Concepts.

Our goal is to enable people to make good decisions * about which claims to believe about the effects of things they can do for their health, the health of others or for other reasons, and about what to do to achieve their goals.

* A good decision is one that makes effective use of the information available to the decision maker at the time the decision is made. A good outcome is one that the decision maker likes. The aim of thinking critically about treatments is to increase the probability of good outcomes (and true conclusions), but many other factors affect outcomes aside from critical thinking (for example genetic and environmental factors). ^70^

We received little feedback on the 2019 version (10 suggestions) and decided that revisions were not needed in 2020 and 2021. ^69^ The 2022 version ^18^ includes the same concepts as the 2019 version. We now provide examples in the explanation for each concept and the basis for each concept, as well as the implications. The 2022 version includes over 600 references, over half of which are to systematic reviews.

We incorporated eight suggestions in the explanations for concepts in this version. For example, a suggestion to include the concept that harms can be irreversible was incorporated in the explanation for the concept “do not assume that treatments are safe” by adding the following to the explanation for that concept: “The harm that is caused may be minor, but treatments also sometimes cause serious, irreversible harms, including death.” ^69^ We also reorganised the concepts into four subgroups (high-level concepts) within each of the first two main groups (claims and comparisons) and into two subgroups within the third main group (choices) ( Table 1). We did this to make the organisation of the concepts more logical and the long list of concepts in some of the subgroups less overwhelming. The 10 high-level concepts also make it easier to get the gist of the concepts and makes the list for some of the subgroups less overwhelming and easier to remember. Table 4 is an overview of the 49 concepts in the three main groups and 10 subgroups.

**Table 4.** Overview of the Informed Health Choices (IHC) Key Concepts. ^18^

1. Claims	2. Comparisons	3. Choices
*Claims about effects that are not supported by evidence from fair comparisons are not necessarily wrong, but there is an insufficient basis for believing them.*	*To identify treatment effects, studies should make fair comparisons, designed to minimize the risk of systematic errors (biases) and random errors (the play of chance).*	*What to do depends on judgements about a problem, the relevance of the available evidence, and the balance of expected benefits, harms, and costs.*
**1.1 Assumptions that treatments are safe or effective can be misleading.** Do not assume that a) treatments are safe, b) treatments have large, dramatic effects, c) treatment effects are certain, d) it is possible to know who will benefit and who will be harmed, or e) comparisons are not needed. **1.2 Seemingly logical assumptions about *research* can be misleading.** Do not assume that a) a plausible explanation is sufficient, b) association is the same as causation, c) more data is better data, d) a single study is sufficient, or e) fair comparisons are not applicable in practice. **1.3 Seemingly logical assumptions about *treatments* can be misleading.** Do not assume that a) treatment is needed, b) more treatment is better, c) a treatment is helpful or safe based on how widely used it is or has been, d) a treatment is better based on how new or technologically impressive it is, or e) earlier detection of ‘disease’ is better. **1.4 Trust based on the source of a claim alone can be misleading.** Do not assume that a) personal experiences alone are sufficient, b) your beliefs are correct, c) opinions alone are sufficient, d) peer review and publication is sufficient, or e) there are no competing interests.	**2.1 Comparisons of treatments should be fair.** Consider whether a) the people being compared were similar, b) the people being compared were cared for similarly, c) the people being compared knew which treatments they received, d) outcomes were assessed similarly in the people being compared, e) outcomes were assessed reliably, f) outcomes were assessed in all (or nearly all) the people being compared, and g) people’s outcomes were analysed in the group to which they were allocated. **2.2 Reviews of the effects of treatments should be fair.** Consider whether a) systematic methods were used, b) unpublished results were considered, c) treatments were compared across studies, and d) important assumptions were tested. **2.3 Descriptions of effects should clearly reflect *the size of the effects.*** Be cautious of a) verbal descriptions alone of the size of effects, b) relative effects of treatments alone, c) average differences between treatments, and d) lack of evidence being interpreted as evidence of “no difference”. **2.4 Descriptions of effects should reflect *the risk of being misled by the play of chance.*** Be cautious of a) small studies, b) results for a selected group of people within a study, c) p-values, and d) results reported as “statistically significant” or “non-significant”.	**3.1 Evidence should be relevant.** a) Be clear about what the problem or goal is and what the options are. Consider the relevance of b) the outcomes measured in the research, c) fair comparisons in laboratories, animals, or highly selected people, d) the treatments that were compared, and e) the circumstances in which the treatments were compared. **3.2 Expected advantages should outweigh expected disadvantages.** a) Weigh the benefits and savings against the harms and costs of acting or not. Consider b) the baseline risk or severity of the symptoms when estimating the size of expected effects, c) how important each advantage and disadvantage is when weighing the pros and cons, d) how certain you can be about each advantage and disadvantage, and e) the need for further fair comparisons.

## Discussion

We made many changes to the IHC Key Concepts after they were first published in 2015. ^35^ The original version included 32 concepts in six groups. There are now 49 concepts in three main groups and 10 subgroups. In addition, there are 20 competences and 15 dispositions. There have been few suggestions for changes to the 2019 version and we have made only minor changes to the explanations for some of the concepts. We therefore have decided that the 2022 version will be the last revision made by us. ^18^ This does not mean that this list of concepts cannot be further improved, but we will leave any further development of the IHC Key Concepts to others.

Although we have attempted to use plain language in describing the key concepts and their basis, the list of key concepts is not intended to be a learning resource for people with no relevant research background. It is a framework, or starting point, for identifying and developing learning resources and evaluation tools. It has proven to be useful for this. ^43^^,^^48^^,^^57^^,^^71^^–^^73^

We have organised the concepts logically. Although it may sometimes make sense to organise learning resources using the same logic, the logic that is used does not reflect the difficulty of the concepts or the order in which the concepts should be learned. Moreover, the full list of concepts can be overwhelming, and it is likely to be necessary to prioritise which concepts to include in learning resources and evaluation tools. ^51^ For example, some concepts are too difficult for primary school children to understand and use, and it may not be possible to incorporate all the concepts in secondary and primary school curricula. ^41^^,^^51^

Ideally, the concepts should be taught and learned using a spiral curriculum, that maps out what students should learn, where they should begin, and how they should progress. ^74^^–^^76^ However, there are many other demands on what to include in primary and secondary school (and other) curricula. ^77^ This is reflected in the findings of a process evaluation of the IHC primary school intervention in Uganda. ^44^ The intervention was shown to have a large effect on primary school children’s ability to think critically about health claims, ^43^ which was sustained after one year. ^45^ Teachers who used the primary school intervention in the trial thought the IHC Key Concepts were important. They were motivated to teach the concepts, and the children were enthusiastic about the lessons. Nonetheless, use of the resources has not been scaled up. A key barrier to scaling up use of the intervention was the need to incorporate the lessons in the national curriculum. The IHC lessons were viewed as an addition to what was already a packed primary school curriculum.

## Conclusions

The IHC Key Concepts framework is central to critical thinking and evidence-based practice, both of which have broader scopes than this framework. ^37^ An important weakness of frameworks with a broader focus is that they usually do not provide an adequate basis (i.e., necessary concepts) for thinking critically about claims about effects and decisions about what to do. The IHC Key Concepts are applicable to a great variety of claims about the effects of interventions, not just health interventions, ^19^ and they are essential points of reference for deciding which claims to believe and what to do.

There is a substantial body of evidence supporting the 49 concepts, as well as logic. Although it is possible to further improve this framework, we will leave any further development of the IHC Key Concepts to others. More importantly, there is a need to incorporate the key concepts into school curricula and to develop, evaluate, and scale up the use of effective educational interventions.

## Data availability

### Underlying data

Zenodo: Suggestions for changes to the IHC Key Concepts 2018-2022. https://doi.org/10.5281/zenodo.6849090. ^69^

The project contains the following underlying data:

• Suggestions for changes to the IHC Key Concepts 2018 – 2022.pdf. (Suggestions for improvements to the 2018 and 2019 versions of the Informed Health Choices Key Concepts and responses).

Data are available under the terms of the Creative Commons Attribution 4.0 International license (CC-BY 4.0).

Zenodo: Key Concepts for assessing claims about treatment effects and making well-informed treatment choices (Version 2022). http://doi.org/10.5281/zenodo.6611932. ^18^

The project contains the following underlying data:

• Informed Health Choices Key Concepts 2022.pdf (Key Concepts for assessing claims about treatment effects and making well-informed treatment choices [Version 2022] - the 2022 version of the Informed Health Choices (IHC) Key Concepts).

Data are available under the terms of the Creative Commons Attribution 4.0 International license (CC-BY 4.0).

## References

[1] Erstad O Voogt J : The twenty-first century curriculum: issues and challenges. Voogt J Knezek G Christensen R , editors. *Second Handbook of Information Technology in Primary and Secondary Education.* Cham, Switzerland: Springer International Publishing;2018. p.19–36. Reference Source

[2] Care E Anderson K Kim H : *Visualizing the breadth of skills movement across education systems.* Washington, DC: Brookings Institution;2016. Reference Source

[3] Voogt J Roblin NP : A comparative analysis of international frameworks for 21st century competences: Implications for national curriculum policies. *J. Curric. Stud.* 2012;44(3):299–321. 10.1080/00220272.2012.668938

[4] Larson L Forzani E Leu DJ : New Literacies: Curricular Implications. Voogt J Knezek G Christensen R , editors. *Second Handbook of Information Technology in Primary and Secondary Education.* Cham, Switzerland: Springer International Publishing;2018; p.19–36. Reference Source

[5] Geng F : A content analysis of the definition of critical thinking. *Asian Soc. Sci.* 2014;10(19):124. 10.5539/ass.v10n19p124

[6] Abrami PC Bernard RM Borokhovski E : Strategies for teaching students to think critically: a meta-analysis. *Rev. Educ. Res.* 2015;85(2):275–314. 10.3102/0034654314551063

[7] Pian W Chi J Ma F : The causes, impacts and countermeasures of COVID-19 “Infodemic”: A systematic review using narrative synthesis. *Inf. Process. Manag.* 2021;58(6):102713. 10.1016/j.ipm.2021.102713 34720340 PMC8545871

[8] Chinn D : Critical health literacy: a review and critical analysis. *Soc Sci Med.* 2011;73(1):60–67. 10.1016/j.socscimed.2011.04.004 21640456

[9] Sykes S Wills J Rowlands G : Understanding critical health literacy: a concept analysis. *BMC Public Health.* 2013;13:150. 10.1186/1471-2458-13-150 23419015 PMC3583748

[10] Chesire F Ochieng M Mugisha M : Contextualizing critical thinking about health using digital technology in secondary schools in Kenya: a qualitative analysis. *Res Square.* 29 March 2022. 10.21203/rs.3.rs-1345080/v1 PMC9535840 36203201

[11] Mugisha M Uwitonze AM Chesire F : Teaching critical thinking about health using digital technology in lower secondary schools in Rwanda: A qualitative context analysis. *PLoS One.* 2021;16(3):e0248773. 10.1371/journal.pone.0248773 33750971 PMC7984628

[12] Ssenyonga R Sewankambo NK Mugagga SK : Learning to think critically about health using digital technology in Ugandan lower secondary schools: a contextual analysis. *PLoS One.* 2022;17(2):e0260367. 10.1371/journal.pone.0260367 35108268 PMC8809610

[13] Lund HM Mathisen PE Rekkavik ME : Teaching critical thinking about health claims: market analysis for Norwegian primary and lower secondary school. *Zenodo.* 2018. 10.5281/zenodo.4748281

[14] Dahlgren A Furuseth-Olsen K Rose CJ : The Norwegian public’s ability to assess treatment claims: results of a cross-sectional study of critical health literacy. *F1000Res.* 2021;9(179). 10.12688/f1000research.21902.2 PMC10995534 38585673

[15] Dewey J : *How We Think.* Boston: DC Heath and Company;1910. Reference Source

[16] Dewey J : The educational significance of concepts. *How We Think: A Restatement of the Relation of Reflective Thinking to the Educative Process.* Boston: DC Heath and Company; 2nd ed. 1933; p.153–4.

[17] Oxman AD Chalmers I Austvoll-Dahlgren A : Informed Health Choices Group. Key Concepts for assessing claims about treatment effects and making well-informed treatment choices. *F1000Res.* 2019;7:1784. 10.12688/f1000research.16771.2 30631443 PMC6290969

[18] Oxman AD Chalmers I Dahlgren A : Informed Health Choices Group. Key Concepts for Informed Health Choices: a framework for enabling people to think critically about health claims (Version 2022). [Dataset]. IHC Working Paper. 2022. 10.5281/zenodo.6611932

[19] Aronson JK Barends E Boruch R : Key concepts for making informed choices. *Nature.* 2019;572(7769):303–306. 10.1038/d41586-019-02407-9 31406318

[20] Chalmers I Oxman AD Austvoll-Dahlgren A : Key Concepts for Informed Health Choices: a framework for helping people learn how to assess treatment claims and make informed choices. *BMJ Evid Based Med.* 2018;23(1):29–33. 10.1136/ebmed-2017-110829 29367324

[21] Chewning B Bylund CL Shah B : Patient preferences for shared decisions: a systematic review. *Patient Educ Couns.* 2012;86(1):9–18. 10.1016/j.pec.2011.02.004 21474265 PMC4530615

[22] Cox K Britten N Hooper R : Patients’ involvement in decisions about medicines: GPs’ perceptions of their preferences. *Br J Gen Pract.* 2007;57(543):777–84. 17925134 PMC2151809

[23] Stacey D Légaré F Col NF : Decision aids for people facing health treatment or screening decisions. *Cochrane Database Syst Rev*. 2017;4(4): CD001431. 10.1002/14651858.CD001431.pub5 PMC6478132 28402085

[24] Walsh T Barr PJ Thompson R : Undetermined impact of patient decision support interventions on healthcare costs and savings: systematic review. *BMJ.* 2014;348: g188. 10.1136/bmj.g188 PMC3900320 24458654

[25] Oxman AD Paulsen EJ : Who can you trust? A review of free online sources of “trustworthy” information about treatment effects for patients and the public. *BMC Med Inform Decis Mak.* 2019;19(1):35. 10.1186/s12911-019-0772-5 30786889 PMC6381637

[26] Glenton C Paulsen EJ Oxman AD : Portals to Wonderland: health portals lead to confusing information about the effects of health care. *BMC Med Inform Decis Mak.* 2005;5:7. 10.1186/1472-6947-5-7 15769291 PMC1079858

[27] Eysenbach G Powell J Kuss O : Empirical studies assessing the quality of health information for consumers on the world wide web: a systematic review. *JAMA.* 2002;287(20):2691–2700. 10.1001/jama.287.20.2691 12020305

[28] Suarez-Lledo V Alvarez-Galvez J : Prevalence of health misinformation on social media: systematic review. *J Med Internet Res.* 2021;23(1): e17187. 10.2196/17187 PMC7857950 33470931

[29] Swire-Thompson B Lazer D : Public health and online misinformation: challenges and recommendations. *Annu Rev Public Health.* 2020;41:433–451. 10.1146/annurev-publhealth-040119-094127 31874069

[30] Borges do Nascimento IJ Pizarro AB Almeida JM : Infodemics and health misinformation: a systematic review of reviews. *Bull World Health Organ*. 2022;100(9):544–561. 10.2471/blt.21.287654 36062247 PMC9421549

[31] World Health Organization : *Declaration of Alma-Ata.* Geneva: World Health Organization;1978. https://apps.who.int/iris/handle/10665/347879

[32] Norheim OF Abi-Rached JM Bright LK : Difficult trade-offs in response to COVID-19: the case for open and inclusive decision making. *Nat Med.* 2021;27(1):10–13. 10.1038/s41591-020-01204-6 33340033

[33] Informed Health Choices Group: The Informed Healthcare Choices Group. Supporting informed healthcare choices in low-income countries – final report. 2018. 10.5281/zenodo.4748333

[34] Evans I Thornton H Chalmers I : *Testing Treatments: Better Research for Better Healthcare.* London: Pinter & Martin; 2nd ed 2011. Reference Source 22171402

[35] Austvoll-Dahlgren A Oxman AD Chalmers I : Key concepts that people need to understand to assess claims about treatment effects. *J. Evid. Based Med.* 2015;8(3):112–125. 10.1111/jebm.12160 26107552

[36] Stewart R Aronson JK Barends E : Lessons from working across fields to develop a framework for informed choices. *Research for All.* 2022;6(1). 10.14324/RFA.06.1.05

[37] Oxman AD Garcia LM : Comparison of the Informed Health Choices Key Concepts Framework to other frameworks relevant to teaching and learning how to think critically about health claims and choices: a systematic review. *F1000Res.* 2020;9:164. 10.12688/f1000research.21858.1 33224475 PMC7670481

[38] Austvoll-Dahlgren A Nsangi A Semakula D : Interventions and assessment tools addressing key concepts people need to know to appraise claims about treatment effects: a systematic mapping review. *Syst. Rev.* 2016;5(1):215. 10.1186/s13643-016-0389-z 28034307 PMC5200965

[39] Cusack L Del Mar CB Chalmers I : Educational interventions to improve people’s understanding of key concepts in assessing the effects of health interventions: a systematic review. *Syst. Rev.* 2018;7(1):68. 10.1186/s13643-018-0719-4 29716639 PMC5930693

[40] Castle JC Chalmers I Atkinson P : Establishing a library of resources to help people understand key concepts in assessing treatment claims-The “Critical thinking and Appraisal Resource Library” (CARL). *PLoS One.* 2017;12(7):e0178666. 10.1371/journal.pone.0178666 28738058 PMC5524286

[41] Nsangi A Semakula D Rosenbaum SE : Development of the informed health choices resources in four countries to teach primary school children to assess claims about treatment effects: a qualitative study employing a user-centred approach. *Pilot Feasibility Stud.* 2020;6:18. 10.1186/s40814-020-00565-6 32055405 PMC7008535

[42] Nsangi A Semakula D Oxman AD : Teaching children in low-income countries to assess claims about treatment effects: prioritization of key concepts. *J. Evid. Based Med.* 2015;8(4):173–180. 10.1111/jebm.12176 26779695

[43] Nsangi A Semakula D Oxman AD : Effects of the Informed Health Choices primary school intervention on the ability of children in Uganda to assess the reliability of claims about treatment effects: a cluster-randomised controlled trial. *Lancet.* 2017;390(10092):374–388. 10.1016/s0140-6736(17)31226-6 28539194

[44] Nsangi A Semakula D Glenton C : Informed health choices intervention to teach primary school children in low-income countries to assess claims about treatment effects: process evaluation. *BMJ Open.* 2019;9(9):e030787. 10.1136/bmjopen-2019-030787 31511291 PMC6747654

[45] Nsangi A Semakula D Oxman AD : Effects of the Informed Health Choices primary school intervention on the ability of children in Uganda to assess the reliability of claims about treatment effects, 1-year follow-up: a cluster-randomised trial. *Trials.* 2020;21(1):27. 10.1186/s13063-019-3960-9 31907013 PMC6945419

[46] Semakula D Nsangi A Oxman M : Development of mass media resources to improve the ability of parents of primary school children in Uganda to assess the trustworthiness of claims about the effects of treatments: a human-centred design approach. *Pilot Feasibility Stud.* 2019;5:155. 10.1186/s40814-019-0540-4 31890267 PMC6935490

[47] Semakula D Nsangi A Oxman AD : Priority setting for resources to improve the understanding of information about claims of treatment effects in the mass media. *J. Evid. Based Med.* 2015;8(2):84–90. 10.1111/jebm.12153 25955161

[48] Semakula D Nsangi A Oxman AD : Effects of the Informed Health Choices podcast on the ability of parents of primary school children in Uganda to assess claims about treatment effects: a randomised controlled trial. *Lancet.* 2017;390(10092):389–398. 10.1016/s0140-6736(17)31225-4 28539196

[49] Semakula D Nsangi A Oxman A : Informed Health Choices media intervention for improving people’s ability to critically appraise the trustworthiness of claims about treatment effects: a mixed-methods process evaluation of a randomised trial in Uganda. *BMJ Open.* 2019;9(12):e031510. 10.1136/bmjopen-2019-031510 31852697 PMC6937069

[50] Rosenbaum S Oxman M Oxman AD : Human-centred design development of Informed Health Choices (IHC) learning resources for secondary school students: protocol. IHC Working Paper. 2019. 10.5281/zenodo.4748445

[51] Agaba JJ Chesire F Mugisha M : Prioritisation of Informed Health Choices (IHC) Key Concepts to be included in lower-secondary school resources: a consensus study. *medRxiv.* 2022. 10.1101/2022.04.11.22273708 PMC10081733 37027357

[52] Semakula D Nsangi A Oxman AD : Effects of the Informed Health Choices podcast on the ability of parents of primary school children in Uganda to assess the trustworthiness of claims about treatment effects: one-year follow up of a randomised trial. *Trials.* 2020;21(1):187. 10.1186/s13063-020-4093-x 32059694 PMC7023790

[53] Ringle VAM : Developing and testing the effects of an educational podcast to improve critical appraisal of healthcare claims. Doctoral dissertation. Miami: University of Miami. 2020.

[54] Austvoll-Dahlgren A Semakula D Nsangi A : Measuring ability to assess claims about treatment effects: the development of the ’Claim Evaluation Tools’. *BMJ Open.* 2017;7(5):e013184. 10.1136/bmjopen-2016-013184 28515181 PMC5777467

[55] Austvoll-Dahlgren A Guttersrud O Nsangi A : Measuring ability to assess claims about treatment effects: a latent trait analysis of items from the ’Claim Evaluation Tools’ database using Rasch modelling. *BMJ Open.* 2017;7(5):e013185. 10.1136/bmjopen-2016-013185 28550019 PMC5777469

[56] Semakula D Nsangi A Oxman AD : Measuring ability to assess claims about treatment effects in English and Luganda: evaluation of multiple-choice questions from the “Claim Evaluation Tools” database using Rasch modelling. 2017. 10.5281/zenodo.4748274 PMC5777469 28550019

[57] Davies A Gerrity M Nordheim L : Measuring ability to assess claims about treatment effects: establishment of a standard for passing and mastery. 2017. 10.5281/zenodo.4748279

[58] Pérez-Gaxiola G Austvoll-Dahlgren A : Psychometric validation of a questionnaire to measure the ability of the public to evaluate claims about treatments. *Gac. Med. Mex.* 2018;154(4):480–495. 10.24875/GMM.17003340 Reference Source 30250337

[59] Wang Q Austvoll-Dahlgren A Zhang J : Evaluating people’s ability to assess treatment claims: Validating a test in Mandarin from Claim Evaluation Tools database. *J. Evid. Based Med.* 2019;12(2):140–146. 10.1111/jebm.12343 31144466

[60] Moberg J Austvoll-Dahlgren A Treweek S : The plain language Glossary of Evaluation Terms for Informed Treatment choices (GET-IT) at www. getitglossary.org. *Research for All.* 2018;2(1):106–121. 10.18546/RFA.02.1.10

[61] Albarqouni L Hoffmann T Straus S : Core competencies in evidence-based practice for health professionals: consensus statement based on a systematic review and Delphi survey. *JAMA Netw. Open.* 2018;1(2):e180281. 10.1001/jamanetworkopen.2018.0281 30646073

[62] Oxman M Larun L Gaxiola GP : Quality of information in news media reports about the effects of health interventions: systematic review and meta-analyses. *F1000Res.* 2022;10:433. 10.12688/f1000research.52894.2 35083033 PMC8756300

[63] Oxman AD Chalmers I Dahlgren A : Key Concepts for assessing claims about treatment effects and making well-informed treatment choices. Version 2019. IHC Working Paper. 2019. 10.5281/zenodo.4746689 PMC6290969 30631443

[64] Clarke M Oxman AD Paulsen E : Guide to the contents of a Cochrane methodology protocol and review. *Cochrane Handbook for Systematic Reviews of Interventions version 5.0.* 2008. Reference Source

[65] Rada G Pérez D Araya-Quintanilla F : Epistemonikos: a comprehensive database of systematic reviews for health decision-making. *BMC Med. Res. Methodol.* 2020;20(1):286. 10.1186/s12874-020-01157-x 33256642 PMC7708132

[66] Austvoll-Dahlgren A Chalmers I Oxman AD : Assessing claims about treatments effects: key concepts that people need to understand (Version 2016). 2016. 10.5281/zenodo.4746689

[67] Austvoll-Dahlgren A Chalmers I Oxman AD : Assessing claims about treatment effects: key concepts that people need to understand (Version 2017). 2017. 10.5281/zenodo.4746689

[68] Oxman AD Chalmers I Austvoll-Dahlgren A : Informed Health Choices Group. Key Concepts for assessing claims about treatment effects and making well-informed treatment choices (Version 2018). 2018. 10.5281/zenodo.4746689 PMC6290969 30631443

[69] Oxman A Chalmers I Dahlgren A : Suggestions for changes to the IHC key concepts 2018-2022 [Dataset]. Zenodo. 2022. 10.5281/zenodo.6849090

[70] Baron J : *Thinking and Deciding.* 4th ed. Cambridge, UK: Cambridge University Press;2008.

[71] Austvoll-Dahlgren A Semakula D Nsangi A : Measuring ability to assess claims about treatment effects: the development of the ’Claim Evaluation Tools’. *BMJ Open.* 2017;7(5): e013184. 10.1136/bmjopen-2016-013184 PMC5777467 28515181

[72] Rosenbaum SE Moberg J Chesire F : Teaching critical thinking about health information and choices in secondary schools: human-centred design of digital resources. *F1000Res*. 2023;12:481. 10.12688/f1000research.132580.1 PMC11377934 39246586

[73] Dahlgren A Semakula D Chesire F : Critical thinking about treatment effects in Eastern Africa: development and Rasch analysis of an assessment tool. *F1000Res*. 2023;12: 887. 10.12688/f1000research.132052.1

[74] Bruner JS : *The Process of Education.* Boston: Harvard University Press;2009.

[75] Harden RM : What is a spiral curriculum?. *Med. Teach.* 1999;21(2):141–143. 10.1080/01421599979752 21275727

[76] Murray JW : Skills development, habits of mind, and the spiral curriculum: A dialectical approach to undergraduate general education curriculum mapping. *Cogent Educ.* 2016;3(1):1156807. 10.1080/2331186X.2016.1156807

[77] Marzano RJ Kendall JS : *A Comprehensive Guide to Designing Standards-Based Districts, Schools, and Classrooms.* Alexandria, VA: Association for Supervision and Curriculum Development;1996. Reference Source

