# Supplementary material for: Key concepts for informed health choices: Where’s the evidence?
Source: F1000Res. 2023 Nov 27;11:890. Originally published 2022 Aug 4. [Version 2] doi: 10.12688/f1000research.123051.2 (PMC10623542; doi:10.12688/f1000research.123051.2)
Supplement: Peer review history

# Reviewer report: Reviewer response for version 2

**DOI:** 10.5256/f1000research.159159.r225740

**Published:** 2024-01-22

**Authors:** Anke Steckelberg

**Affiliations:** 1 Interdisciplinary Center for Health Sciences, Institute of Health and Nursing Science, Martin Luther University, Halle, Germany

Thank you. I have no further comments.

Is the work clearly and accurately presented and does it cite the current literature?

Yes

If applicable, is the statistical analysis and its interpretation appropriate?

Not applicable

Are all the source data underlying the results available to ensure full reproducibility?

Partly

Is the study design appropriate and is the work technically sound?

Yes

Are the conclusions drawn adequately supported by the results?

Yes

Are sufficient details of methods and analysis provided to allow replication by others?

Partly

Reviewer Expertise:

Evidence based health information; informed decision making; critical health literacy

I confirm that I have read this submission and believe that I have an appropriate level of expertise to confirm that it is of an acceptable scientific standard.

# Reviewer report: Reviewer response for version 1

**DOI:** 10.5256/f1000research.135115.r146595

**Published:** 2023-11-01

**Authors:** Barbara Gasteiger-Klicpera, Michaela Wright

**Affiliations:** 1 Inclusive Education Unit, Institute of Professional Development in Education, University of Graz, Graz, Austria; 2 Research Center for Inclusive Education (RCIE), Institute of Education Research and Teacher Education, University of Graz, Graz, Austria

In general, the paper is easy to follow and well-structured. The objectives are clear, and methods and results are reported in a precise and sound manner. The high degree of transparency is especially notable. Here are a few minor suggestions for clarification:

- a more substantive description (overview) of the key concepts would be helpful 
- p3, 2 ^nd^ paragraph: use “competences” instead of “abilities” for consistent use of language and describe both briefly, as they are integral parts of the framework
- p3, 3 ^rd^ paragraph: “A major new challenge […]” – to what?
- p4: If all of p4 refers to prior revisions, the suggestion is to include the time frame in the heading, e.g.: “Development of the Informed Health Choices Key Concepts from 2013-2018”
- These two points created confusion:
  - p4, 2 ^nd^ paragraph: the cited works from colleagues were published after 2018 (Aronson *et al.,* 2019 and Stewart *et al., *2022)
  - p4, 3 ^rd^ paragraph: the given reference was published in 2020
- p5, 1 ^st^ paragraph: refer to the supplemental material for underlying data within the text
- p6, table 2: include a description of the added competences and disposition in the table (like was done for the added concepts); or provide an explanation why they are not listed; or add reference to where they may be found
- p7, table 3: “[…] many other factors affect outcomes […]” – this wording sparks curiosity about those other factors and weakens the importance of critical thinking
- p8, 2 ^nd^ paragraph: provide an example (within the text) of a suggestion that was incorporated in the explanations for concepts
- p8, 4 ^th^ paragraph: “It has proven to be useful for this.” – missing citation
- Rethink repetitions: “We received little feedback […]” (p8, 1 ^st^ paragraph); “There have been few suggestions for change […]” (p8, 3 ^rd^ paragraph); “We have received few suggestions […]” (p9, 2 ^nd^ paragraph)
- p9, data availability: The suggestion is to add the following to the 2 ^nd^ sentence: The project contains the following underlying data “in two different formats (pdf, docx)”. As the files have different names, the addition makes it clearer that they contain the same information.

Is the work clearly and accurately presented and does it cite the current literature?

Yes

If applicable, is the statistical analysis and its interpretation appropriate?

Not applicable

Are all the source data underlying the results available to ensure full reproducibility?

Yes

Is the study design appropriate and is the work technically sound?

Yes

Are the conclusions drawn adequately supported by the results?

Yes

Are sufficient details of methods and analysis provided to allow replication by others?

Yes

Reviewer Expertise:

Educational research; Educational psychology; Inclusive education; Orthopaedagogy; Child and adolescent psychology;

We confirm that we have read this submission and believe that we have an appropriate level of expertise to confirm that it is of an acceptable scientific standard.

# Response

**Published:** 2023-11-20

**Authors:** Andrew David Oxman

**Affiliations:** Norwegian Institute of Public Health, Norway

Thank you for your comments. Please find our responses below.

1. a more substantive description (overview) of the key concepts would be helpful  
**Response**: We have added the following to the Introduction:
  The concepts serve as the basis for developing learning resources to help people understand and apply the concepts when claims about the effects of health actions are made, and when they make health choices. ^48^ They are also the basis for an item bank of multiple-choice questions (the Claim Evaluation Tools item bank) that can be used for assessing people’s ability to apply the IHC Key Concepts. ^77^
  The concepts are principles for recognising when a claim about the effects of health actions has an untrustworthy basis, recognising when evidence from comparisons of health actions is trustworthy and when it is not, and making well-informed health choices. They can help anyone, not just researchers, to think critically about whether to believe a claim and what to do. The concepts are intended for people using research, not for doing research.
  The concepts and the basis for the concepts tend to focus on ways in which claims and comparisons (evidence) can be misleading, and choices can be misinformed. This is not because we underestimate the tremendous gains that have been made in health care based on appropriate use of research. Our aim is to promote healthy scepticism, not excessive scepticism, and to help people decide what to believe and what to do. How sure we can be about the effects of health actions varies, as does how sure we can be about the balance between the advantages and disadvantages of health actions. When teaching, learning, or using these concepts, it is important to bear this in mind.
**77** Austvoll-Dahlgren A, Semakula D, Nsangi A, Oxman AD, Chalmers I, Rosenbaum S, et al. Measuring ability to assess claims about treatment effects: the development of the 'Claim Evaluation Tools'. *BMJ Open*. 2017;7(5):e013184. 10.1136/bmjopen-2016-013184
2. p3, 2 ^nd^ paragraph: use “competences” instead of “abilities” for consistent use of language and describe both briefly, as they are integral parts of the framework
**Response**: We changed abilities to competences in this paragraph.
3. p3, 3 ^rd^ paragraph: “A major new challenge […]” – to what?
**Response**:  We have edited the sentence to read:
  A major new challenge to deciding what to believe or do is increased access to information online and in social media, and the need to evaluate that information, much of which is misinformation.
4. p4: If all of p4 refers to prior revisions, the suggestion is to include the time frame in the heading, e.g.: “Development of the Informed Health Choices Key Concepts from 2013-2018”
**Response**: We have added 2013-2018 to the heading.
5. These two points created confusion:
  - p4, 2 ^nd^ paragraph: the cited works from colleagues were published after 2018 (Aronson *et al.,* 2019 and Stewart *et al., *2022)
  - p4, 3 ^rd^ paragraph: the given reference was published in 2020
**Response**: There was, as is typical, a delay between when the work was done and when it was published. We have edited the first sentence to read:
  Subsequently, we collected feedback at a series of workshops in 2017 and 2018, ^17^ and from colleagues working in other fields besides health in 2018. ^19,23^
  We have edited the second sentence to read:
  In addition, revisions have been informed by a review of related frameworks conducted in 2018, ^24^
6. p5, 1 ^st^ paragraph: refer to the supplemental material for underlying data within the text
**Response**: We don’t understand this comment. There is not relevant underlying data for the first paragraph on page 5. The underlying data for this paper is cited in the first paragraph of the Methods section (the second paragraph on page 5).
7. p6, table 2: include a description of the added competences and disposition in the table (like was done for the added concepts); or provide an explanation why they are not listed; or add reference to where they may be found
**Response**: We have added the following footnote to the table:
  *The competences and dispositions were reformulated and reorganised, as well as expanded.
8. p7, table 3: “[…] many other factors affect outcomes […]” – this wording sparks curiosity about those other factors and weakens the importance of critical thinking
**Response**: We have edited the sentence to read:
  The aim of thinking critically about treatments is to increase the probability of good outcomes (and true conclusions), but many other factors affect outcomes aside from critical thinking (for example genetic and environmental factors). ^58^
9. p8, 2 ^nd^ paragraph: provide an example (within the text) of a suggestion that was incorporated in the explanations for concepts
**Response**: We added the following sentence to this paragraph:
  For example, a suggestion to include the concept that harms can be irreversible was incorporated in the explanation for the concept “do not assume that treatments are safe” by adding the following to the explanation for that concept: "The harm that is caused may be minor, treatments also sometimes cause serious, irreversible harms, including death.”
10. p8, 4 ^th^ paragraph: “It has proven to be useful for this.” – missing citation
**Response**: We have added citations for the following references:
**77** Austvoll-Dahlgren A, Semakula D, Nsangi A, Oxman AD, Chalmers I, Rosenbaum S, et al. Measuring ability to assess claims about treatment effects: the development of the 'Claim Evaluation Tools'. *BMJ Open*. 2017;7(5):e013184. 10.1136/bmjopen-2016-013184
**78** Nsangi A, Semakula D, Rosenbaum SE, Oxman AD, Oxman M, Morelli A, et al. Development of the informed health choices resources in four countries to teach primary school children to assess claims about treatment effects: a qualitative study employing a user-centred approach. *Pilot Feasibility Stud*. 2020;6:18. 10.1186/s40814-020-00565-6
**79** Semakula D, Nsangi A, Oxman M, Rosenbaum SE, Oxman AD, Austvoll-Dahlgren A, et al. Development of mass media resources to improve the ability of parents of primary school children in Uganda to assess the trustworthiness of claims about the effects of treatments: a human-centred design approach. *Pilot Feasibility Stud*. 2019;5:155. 10.1186/s40814-019-0540-4
**80** Rosenbaum SE, Moberg J, Chesire F, Mugisha M, Ssenyonga R, Ochieng M, et al. Teaching critical thinking about health information and choices in secondary schools: human-centred design of digital resources. *F1000Res*. 2023;12:481. 10.12688/f1000research.132580.1
**81** Austvoll-Dahlgren A, Guttersrud O, Nsangi A, Semakula D, Oxman AD, Group IHC. Measuring ability to assess claims about treatment effects: a latent trait analysis of items from the 'Claim Evaluation Tools' database using Rasch modelling. *BMJ Open*. 2017;7(5):e013185. 10.1136/bmjopen-2016-013185
**82** Dahlgren A, Semakula D, Chesire F, Oxman AD, Mugisha M, Nakyejwe E, et al. Critical thinking about treatment effects in Eastern Africa: development and Rasch analysis of an assessment tool. *F1000Res*. 2023;12:887. 10.12688/f1000research.132052.1
11. Rethink repetitions: “We received little feedback […]” (p8, 1 ^st^ paragraph); “There have been few suggestions for change […]” (p8, 3 ^rd^ paragraph); “We have received few suggestions […]” (p9, 2 ^nd^ paragraph)
**Response**: We removed this sentence from the Conclusions: 
  We have received few suggestions for changes to the 2019 version of the IHC Key Concepts, and earlier versions of the framework have proven to be useful for developing and evaluating educational interventions to help people make good decisions about which claims to believe and what to do.
12. p9, data availability: The suggestion is to add the following to the 2 ^nd^ sentence: The project contains the following underlying data “in two different formats (pdf, docx)”. As the files have different names, the addition makes it clearer that they contain the same information.
**Response**: We deleted the reference to the Word document.

# Reviewer report: Reviewer response for version 1

**DOI:** 10.5256/f1000research.135115.r175378

**Published:** 2023-10-31

**Authors:** Paulo Nadanovsky

**Affiliations:** 1 Department of Epidemiology, Institute of Social Medicine, Rio de Janeiro State University, Rio de Janeiro, Brazil; 2 Department of Epidemiology, National School of Public Health, Oswaldo Cruz Foundation, Rio de Janeiro, Brazil

The Informed Health Choices (IHC) is an important initiative to promote scientific and health literacy. To maintain its effectiveness, it is crucial for the authors to actively engage in updating the IHC. The approach they have chosen, which involves combining literature searches that prioritize systematic reviews and incorporating feedback from experts, is commendable. This strategy guarantees the ongoing maintenance and relevance of the IHC.

 In the introduction, the authors presented a justification for the IHC initiative, stating that "many people find it difficult to make decisions about what to believe or do regarding treatments or health actions." Typically, individuals rely on their physicians to make these judgments on their behalf. People often feel that it is not their responsibility to make decisions about treatments or health actions. It may be beneficial for the authors to address this point in the introduction to alleviate any concerns readers may have.

 In Table 1, the concept presented in item 1.4 states, "Trust based on the source of a claim alone can be misleading." However, this concept poses some challenges. Considering the vast array of expertise in various fields, it is unrealistic for anyone to be able to critically evaluate the validity of every significant claim. For example, personally, I find it difficult to assess claims related to climate change, relying instead on trust in certain sources. In light of this, perhaps the IHC initiative could offer guidance to help individuals discern which sources of information are more trustworthy, rather than emphasizing that trust should not be based solely on the source of a claim.

Is the work clearly and accurately presented and does it cite the current literature?

Yes

If applicable, is the statistical analysis and its interpretation appropriate?

Not applicable

Are all the source data underlying the results available to ensure full reproducibility?

Yes

Is the study design appropriate and is the work technically sound?

Yes

Are the conclusions drawn adequately supported by the results?

Yes

Are sufficient details of methods and analysis provided to allow replication by others?

Yes

Reviewer Expertise:

Public Health; Epidemiology; Evidence-Based Practice; Systematic Review.

I confirm that I have read this submission and believe that I have an appropriate level of expertise to confirm that it is of an acceptable scientific standard.

# Response

**Published:** 2023-11-20

**Authors:** Andrew David Oxman

**Affiliations:** Norwegian Institute of Public Health, Norway

Thank you for your comments. Please find our responses below.

1. In the introduction, the authors presented a justification for the IHC initiative, stating that "many people find it difficult to make decisions about what to believe or do regarding treatments or health actions." Typically, individuals rely on their physicians to make these judgments on their behalf. People often feel that it is not their responsibility to make decisions about treatments or health actions. It may be beneficial for the authors to address this point in the introduction to alleviate any concerns readers may have.
**Response**: We have added the following to the Introduction:
  Although most people prefer to make medical decisions together with a health professional, some people prefer to delegate decisions to a health professional. ^63^ Unfortunately, health professionals’ perceptions of their patients’ desire to be involved in decisions are often inaccurate. ^64^ They may be more likely to underestimate the extent to which patients prefer to be involved in decisions. Regardless of who decides, decisions should be consistent with a patient’s values. Decision aids can help patients to clarify their values and may help them to make choices that are more consistent with their values compared to choices made without decision aids. ^65^ There is some evidence that patients choose more conservative approaches when they become better informed. ^66^
  Even people who prefer to delegate medical decisions to a health professional are confronted with an over-abundance of information about things they can do for their health, much of which is unreliable. ^67-73^ Although much of this information can simply be ignored, to avoid being misled by claims with an untrustworthy basis, people need to be able to recognize claims about the effects of health actions, question the basis for those claims, and recognize when a claim is relevant and important, and warrants reflection.
  People have the right to participate collectively as well as individually in the planning and implementation of their health care. ^74^ In addition to being a democratic right, public participation in deliberative and decision-making processes has the potential to improve the quality of the judgements and decisions that are made, build trust, improve adherence, and help to ensure transparency and accountability. ^75^ However, the extent to which potential benefits of public participation are achieved (and potential harms are avoided) depends, among other things, on the ability of citizens to think critically about collective decisions as well as personal choice.
**63** Chewning B, Bylund CL, Shah B, Arora NK, Gueguen JA, Makoul G. Patient preferences for shared decisions: a systematic review. *Patient Educ. Couns*. 2012;86(1):9-18. 10.1016/j.pec.2011.02.004
**64** Cox K, Britten N, Hooper R, White P. Patients' involvement in decisions about medicines: GPs' perceptions of their preferences. *Br. J. Gen. Pract.* 2007;57(543):777-84. PMCID: PMC2151809
**65** Stacey D, Légaré F, Col NF, Bennett CL, Barry MJ, Eden KB, et al. Decision aids for people facing health treatment or screening decisions. *Cochrane Database Syst. Rev*. 2017;4(4):CD001431. 10.1002/14651858.CD001431.pub5
**66** Walsh T, Barr PJ, Thompson R, Ozanne E, O'Neill C, Elwyn G. Undetermined impact of patient decision support interventions on healthcare costs and savings: systematic review. *BMJ*. 2014;348:g188. 10.1136/bmj.g188
**67** Oxman AD, Paulsen EJ. Who can you trust? A review of free online sources of "trustworthy" information about treatment effects for patients and the public. BMC *Med. Inform. Decis. Mak*. 2019;19(1):35. 10.1186/s12911-019-0772-5
**68** Glenton C, Paulsen EJ, Oxman AD. Portals to Wonderland: health portals lead to confusing information about the effects of health care. *BMC Med. Inform. Decis. Mak*. 2005;5:7. https://doi.org/10.1186/1472-6947-5-7
**69** Eysenbach G, Powell J, Kuss O, Sa ER. Empirical studies assessing the quality of health information for consumers on the world wide web: a systematic review. *JAMA*. 2002;287(20):2691-700. 10.1001/jama.287.20.2691
**70** Suarez-Lledo V, Alvarez-Galvez J. Prevalence of health misinformation on social media: systematic review. *J. Med. Internet. Res*. 2021;23(1):e17187. 10.2196/17187
**71** Swire-Thompson B, Lazer D. Public health and online misinformation: challenges and recommendations. *Annu. Rev. Public Health*. 2020;41:433-51. 10.1146/annurev-publhealth-040119-094127
**72** Pian W, Chi J, Ma F. The causes, impacts and countermeasures of COVID-19 "Infodemic": A systematic review using narrative synthesis. *Inf. Process. Manag*. 2021;58(6):102713. 10.1016/j.ipm.2021.102713
**73** Borges do Nascimento IJ, Pizarro AB, Almeida JM, Azzopardi-Muscat N, Gonçalves MA, Björklund M, et al. Infodemics and health misinformation: a systematic review of reviews. *Bull. World Health Organ*. 2022;100(9):544-61. 10.2471/blt.21.287654
**74** World Health Organization. Declaration of Alma-Ata. Geneva: World Health Organization; 1978. https://apps.who.int/iris/handle/10665/347879
**75** Norheim OF, Abi-Rached JM, Bright LK, Bærøe K, Ferraz OLM, Gloppen S, et al. Difficult trade-offs in response to COVID-19: the case for open and inclusive decision making. *Nat. Med*. 2021;27(1):10-3. 10.1038/s41591-020-01204-6
2. In Table 1, the concept presented in item 1.4 states, "Trust based on the source of a claim alone can be misleading." However, this concept poses some challenges. Considering the vast array of expertise in various fields, it is unrealistic for anyone to be able to critically evaluate the validity of every significant claim. For example, personally, I find it difficult to assess claims related to climate change, relying instead on trust in certain sources. In light of this, perhaps the IHC initiative could offer guidance to help individuals discern which sources of information are more trustworthy, rather than emphasizing that trust should not be based solely on the source of a claim.
**Response**: We have added this paragraph to the Introduction:
  People often disagree about the effects of health actions, including experts. Who makes a claim, how likable they are, or how much experience and expertise they have do not provide a reliable basis for assessing how reliable their claim is. This does not mean that conflicting opinions should be given equal weight – or that the existence of conflicting opinions means that no conclusion can be reached. It also does not mean that all sources of information are equally reliable. How much weight to give an opinion should be based on the strength of the evidence supporting it. How much trust to place in a source of information about health actions should be based on whether it provides information about the effects of health actions that is based on systematic reviews. ^76^
**76** Oxman AD, Paulsen EJ. Who can you trust? A review of free online sources of "trustworthy" information about treatment effects for patients and the public. BMC *Med. Inform. Decis. Mak*. 2019;19(1):35. 10.1186/s12911-019-0772-5

# Reviewer report: Reviewer response for version 1

**DOI:** 10.5256/f1000research.135115.r146596

**Published:** 2022-09-01

**Authors:** Anke Steckelberg

**Affiliations:** 1 Interdisciplinary Center for Health Sciences, Institute of Health and Nursing Science, Martin Luther University, Halle, Germany

Thank you for giving me the chance to review your manuscript. Please find my comments below:

1. The authors reported that they responded to feedback on the 2018 version to finalise the 2019 version. It remains unclear who was asked to give feedback? How many of those who were asked to give feedback, finally gave feedback? Please provide additional information.
2. The authors searched for reviews to provide a basis for the concepts. Whenever possible, they included systematic reviews. The authors also mentioned reviews. What designs were included? Please clarify.
3. The conclusion needs to be revised. It partly repeats the results. 

Is the work clearly and accurately presented and does it cite the current literature?

Yes

If applicable, is the statistical analysis and its interpretation appropriate?

Not applicable

Are all the source data underlying the results available to ensure full reproducibility?

Partly

Is the study design appropriate and is the work technically sound?

Yes

Are the conclusions drawn adequately supported by the results?

Yes

Are sufficient details of methods and analysis provided to allow replication by others?

Partly

Reviewer Expertise:

Evidence based health information; informed decision making; critical health literacy

I confirm that I have read this submission and believe that I have an appropriate level of expertise to confirm that it is of an acceptable scientific standard, however I have significant reservations, as outlined above.

# Response

**Published:** 2023-11-20

**Authors:** Andrew David Oxman

**Affiliations:** Norwegian Institute of Public Health, Norway

Thank you for your comments. Please find our responses below.

1. The authors reported that they responded to feedback on the 2018 version to finalize the 2019 version. It remains unclear who was asked to give feedback? How many of those who were asked to give feedback, finally gave feedback? Please provide additional information.
**Response**: We have added the following to the Methods section:
  The following information was included in the 2018 and 2019 versions: Please send any comments or suggestions to: contact@informedhealthchoices.org. In addition, we asked people to provide feedback at workshops where we presented the Key Concepts. Seven individuals provided feedback on the 2018 version and four provided feedback on the 2019 version. The suggestions came from members of the IHC Network and other researchers.
2. The authors searched for reviews to provide a basis for the concepts. Whenever possible, they included systematic reviews. The authors also mentioned reviews. What designs were included? Please clarify.
**Response**: We have added the following to the Methods section:
  We included systematic reviews of methodological studies and overviews of reviews, such as Cochrane methodology reviews, methodological studies based on a systematic review of research (“meta-epidemiological” studies), and systematic reviews of treatment effects. We categorized as systematic reviews any review of research that included a methods section that described the search strategy for finding studies and selection criteria.
3. The conclusion needs to be revised. It partly repeats the results. 
**Response**: We have deleted the following sentence from the Conclusions:
  We have received few suggestions for changes to the 2019 version of the IHC Key Concepts, and earlier versions of the framework have proven to be useful for developing and evaluating educational interventions to help people make good decisions about which claims to believe and what to do.
